# Case Report: Complete response after tislelizumab treatment in a hepatocellular carcinoma patient with abdominal lymph node metastasis

**DOI:** 10.3389/fimmu.2023.1163656

**Published:** 2023-04-25

**Authors:** Haihui Deng, Bin Chen, Deti Peng, Jian He, Weicheng Zhao, Tuantuan Chen, Zonggui Xie, Fuwen Pang

**Affiliations:** ^1^ Department of Interventional Radiology, Shenzhen Traditional Chinese Medicine Hospital, Shenzhen, China; ^2^ Department of Liver Disease, Shenzhen Traditional Chinese Medicine Hospital, Shenzhen, China

**Keywords:** hepatocellular carcinoma, immunotherapy, locoregional therapy, tislelizumab, case report

## Abstract

**Background:**

Abdominal lymph node (ALN) metastasis is associated with a poor prognosis in patients with hepatocellular carcinoma (HCC) because of the limited number of effective therapeutic options available. Immunotherapy with immune checkpoint inhibitors, such as those targeting programmed death receptor-1 (PD-1), have produced encouraging results in patients with advanced HCC. Here, we report a complete response (CR) in a patient with advanced HCC and ALN metastasis after combination treatment with tislelizumab (a PD-1 inhibitor) and locoregional therapy.

**Case summary:**

A 58-year-old man with HCC experienced progressive disease with multiple ALN metastases after undergoing transcatheter arterial chemoembolization (TACE), radiofrequency ablation (RFA), and laparoscopic resection. Because the patient did not wish to receive systemic therapy, including chemotherapy and targeting therapy, we prescribed tislelizumab (as a single immunotherapeutic agent) together with RFA. After four tislelizumab treatment cycles, the patient achieved a CR without tumor recurrence for up to 15 months.

**Conclusion:**

Tislelizumab monotherapy can be effectively used to treat advanced HCC with ALN metastasis. Moreover, the combination of locoregional therapy and tislelizumab is likely to further increase therapeutic efficacy.

## Introduction

Abdominal lymph nodes (ALNs) are one of the most common sites of extrahepatic metastatic hepatocellular carcinoma (HCC) (1), which is associated with a poor prognosis (2). Longer survival may be possible with effective treatment of ALN metastases. ALN metastases in HCC are routinely treated using surgical resection, external radiotherapy, and chemotherapy. However, surgical resection of ALNs, and especially retroperitoneal LNs, is challenging due to their hard to read location and close proximity to vital organs such as the vascular system, biliary tract, and gastrointestinal tracts (3). External radiotherapy has proven to be effective in shrinking the tumor and relieving symptoms. However, it is not curative and is usually limited by the poor tolerance of the surrounding normal tissues or organs to treatment (3, 4). In addition, the low chemosensitivity of HCC and the generally poor conditions of patients with HCC recurrence hinder the feasibility of chemotherapy (5, 6). Therefore, the question of which treatment strategy is most effective for use in patients with ALN metastatic HCC is under debate, with current treatment strategies often based on individual experience.

Immune checkpoint inhibitors (ICIs) are promising therapeutic agents for inhibiting HCC tumor progression, recurrence, and metastasis (7). Tislelizumab is a humanized IgG4 monoclonal antibody with a high affinity and specificity for programmed cell death-1 (PD-1). Tislelizumab was approved for the treatment of HCC by the China National Medical Product Administration and the U.S. Food and Drug Administration (FDA) (8, 9). Here, we report a case of HCC with ALN metastases, whereby the patient achieved a CR and remained in remission 15 months after tislelizumab treatment initiation.

## Case report

**Figure 1:**
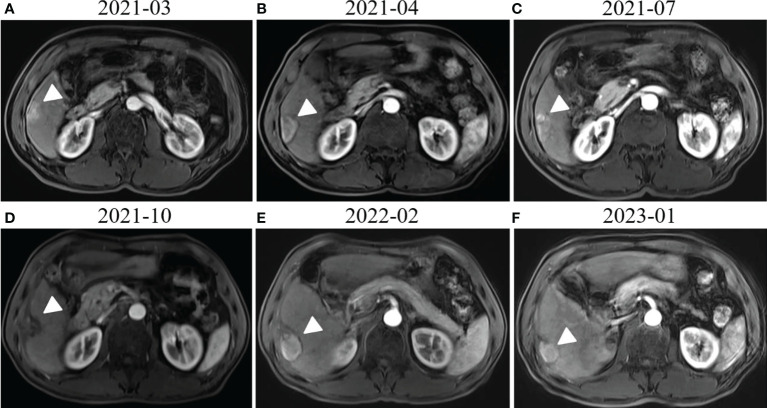
Radiological response evaluation of the liver during the clinical course. **(A)** Contrast-enhanced magnetic resonance imaging (MRI) revealed enhanced subcapsular hepatocellular carcinoma (HCC) lesions in the hepatic S6 (white arrowhead). **(B)** Contrast-enhanced MRI revealed that the lesion in hepatic S6 was not enhanced in the arterial phase (white arrowhead) 1 month after transcatheter arterial chemoembolization (TACE) and radiofrequency ablation (RFA). **(C)** An enhanced small nodule around the previous one (white arrowhead) was detected by MRI three months after TACE and RFA. **(D)** Contrast-enhanced MRI showed no active lesions in the liver (white arrowhead) 3 months after surgical resection of the liver metastases. **(E)** Complete response (CR) was confirmed by contrast-enhanced MRI, which showed no active lesions in the liver (white arrowhead) after RFA and four cycles of tislelizumab treatment. **(F)** MRI obtained 15 months after tislelizumab treatment initiation showed that there were still no active lesions (white arrowhead) in the liver or new lesion.

**Figure 2:**
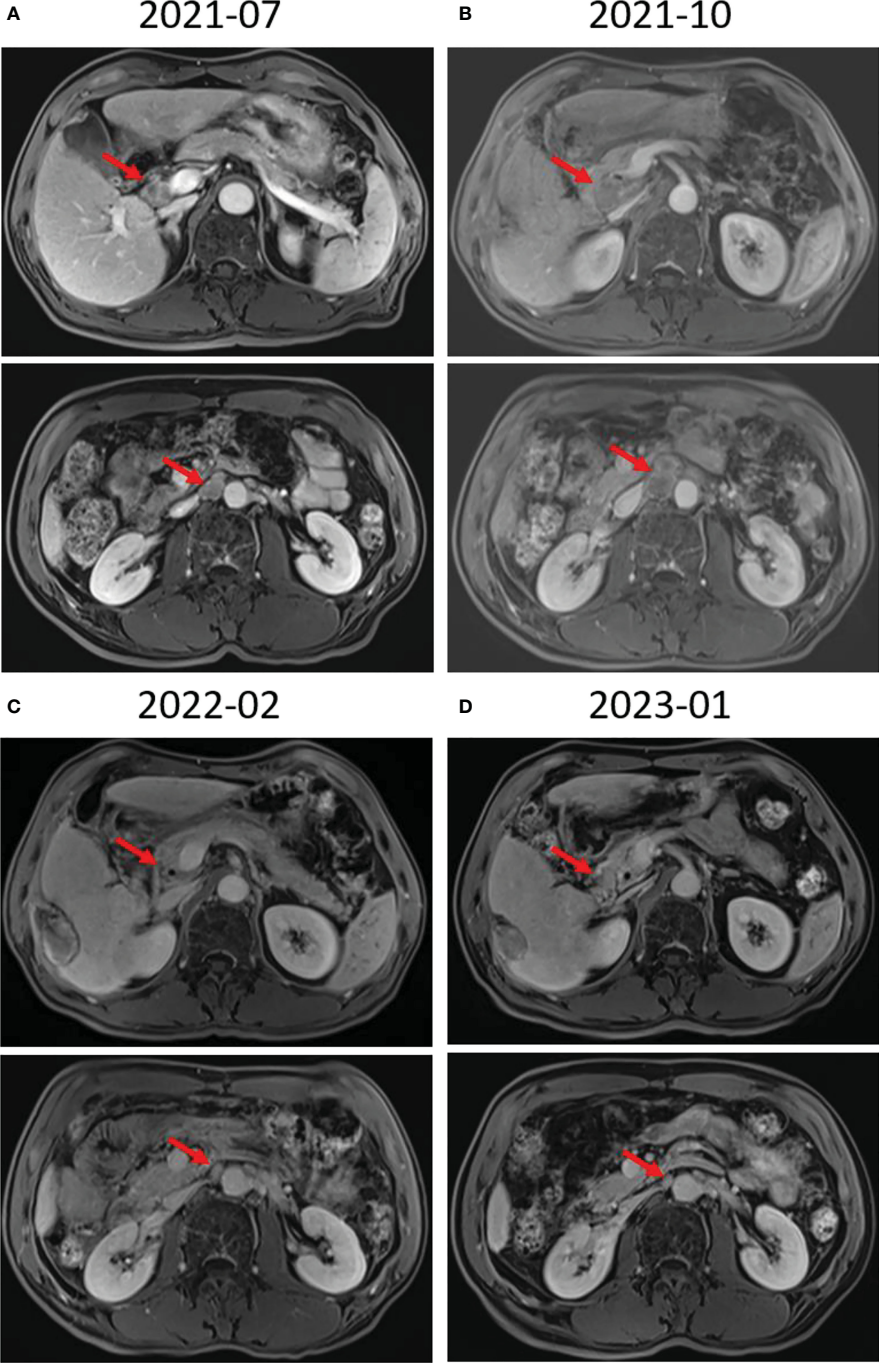
Radiological response evaluation of abdominal lymph nodes during the clinical course. **(A)** Contrast-enhanced magnetic resonance imaging (MRI) revealed multiple enlarged abdominal lymph nodes (red arrow). **(B)** Contrast-enhanced MRI showed that there were still multiple enlarged abdominal lymph nodes (red arrow) 3 months after surgical resection of lymph nodes in the hilar area. **(C)** Contrast-enhanced MRI showed that the size of the abdominal lymph nodes was reduced after RFA and four cycles of tislelizumab treatment (red arrow). **(D)** A significant reduction in the size of the abdominal lymph nodes (≤10 mm) was seen 15 months after tislelizumab treatment initiation (red arrow).

**Figure 3:**
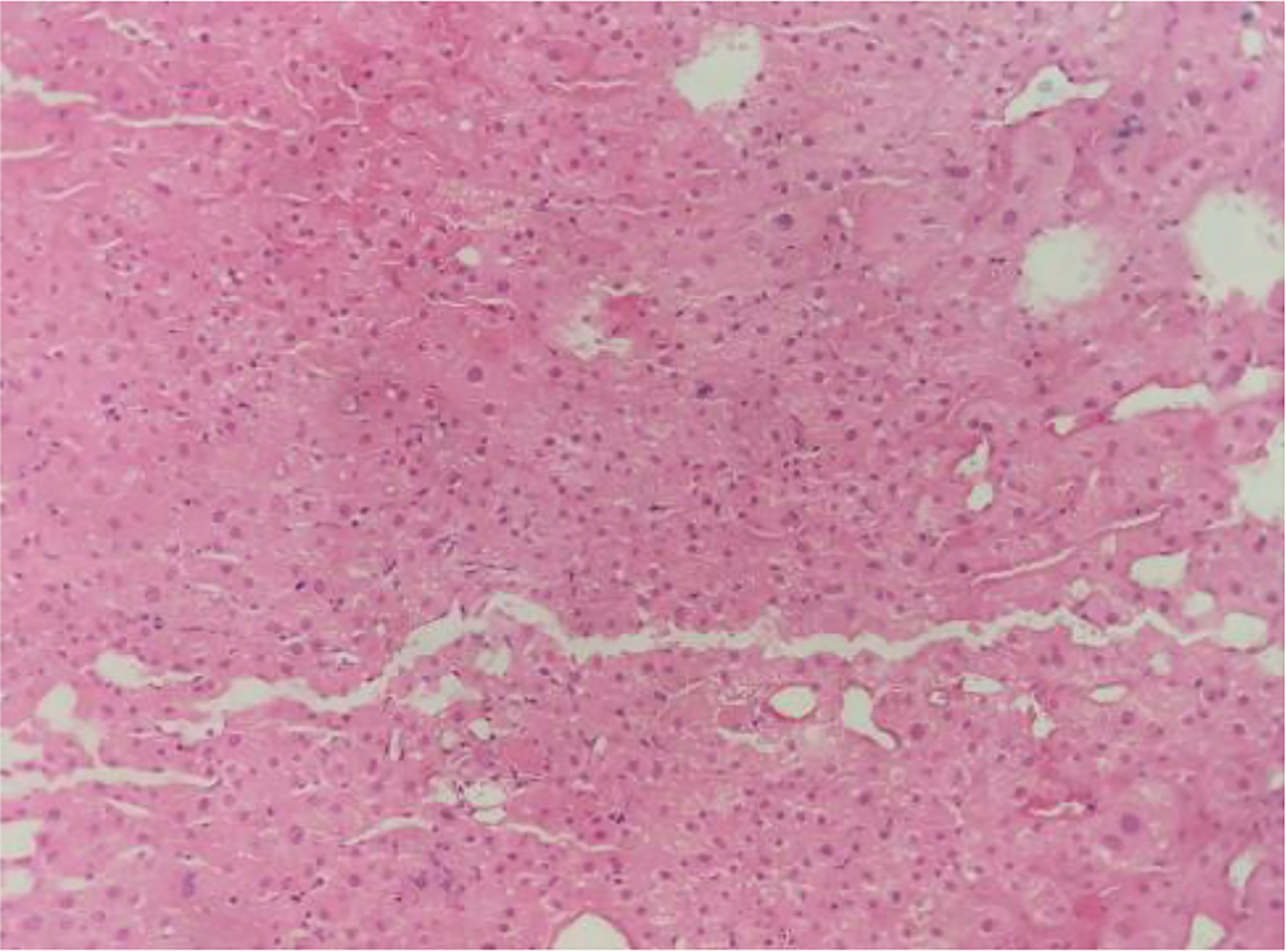
Histological findings of the liver at autopsy (hematoxylin and eosin staining). The pathological diagnosis is hepatocellular carcinoma.

**Figure 4:**
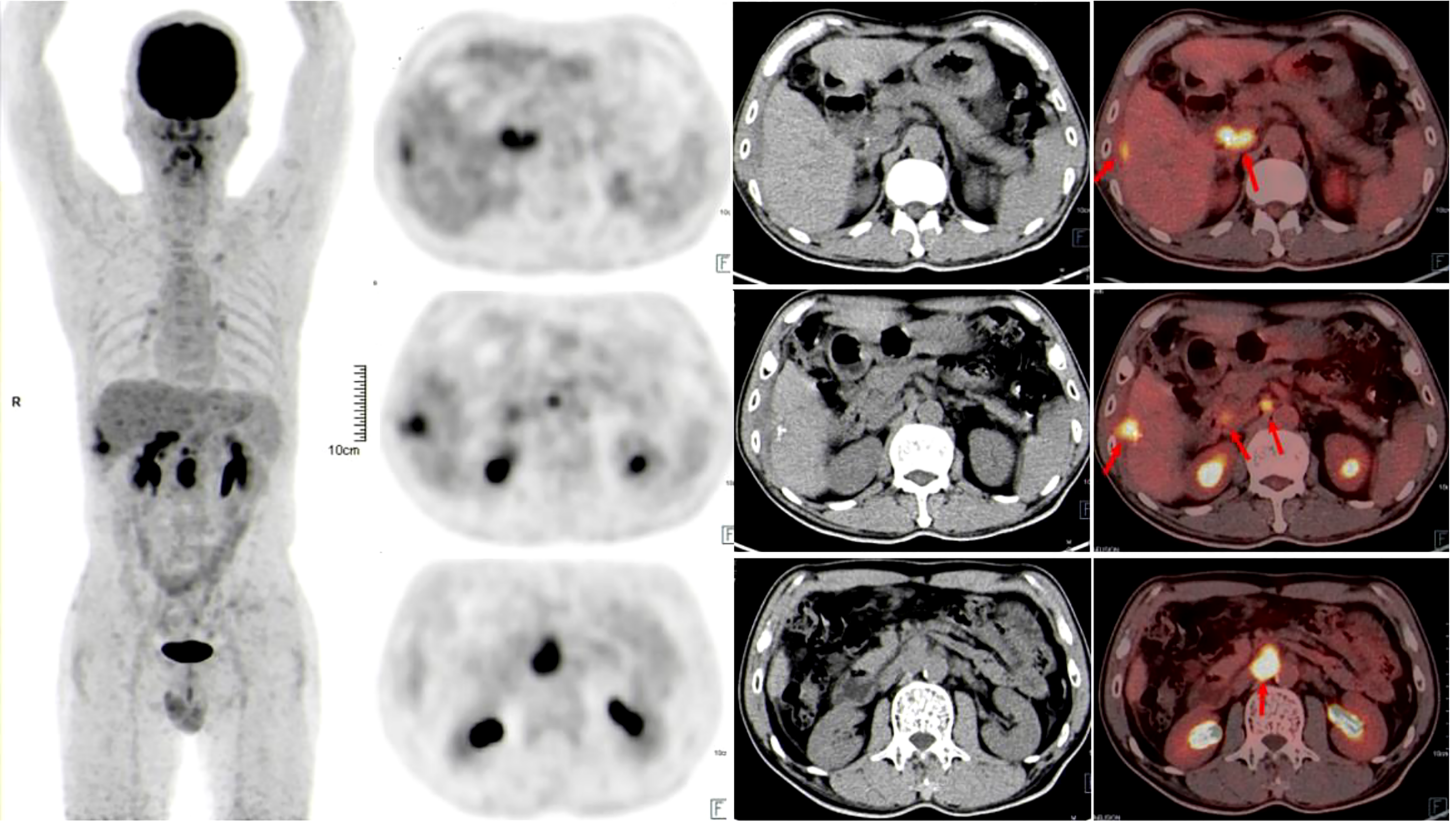
Positron emission tomography-computer tomography showed that glucose metabolism was significantly increased in the liver and abdominal lymph node lesions 3 months after surgical resection of HCC lesions and lymph nodes in the hilar area (red arrows).

**Figure 5:**
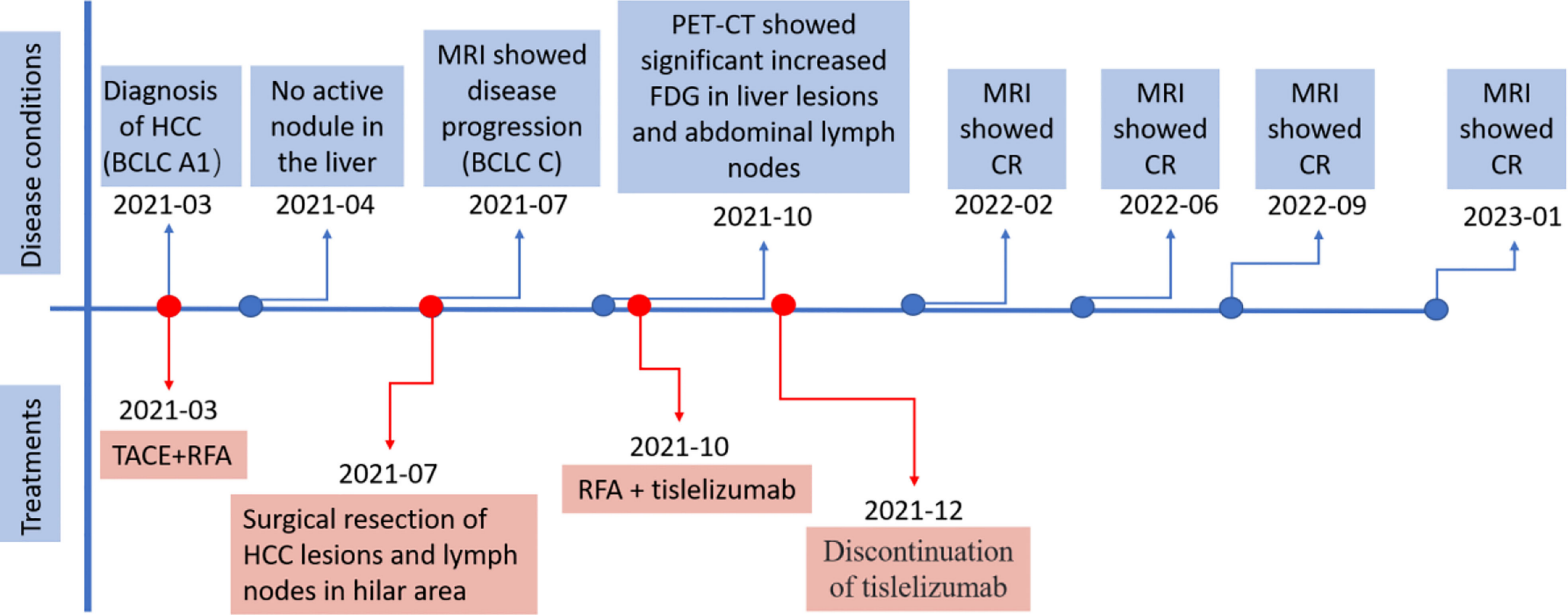
The treatment timeline. HCC, hepatocellular carcinoma; BCLC, Barcelona Clinic Liver Cancer. TACE, transcatheter arterial chemoembolization; RFA, radiofrequency ablation; MRI, magnetic resonance imaging; PET-CT, positron emission tomography-computer tomography; FDG, fluorodeoxyglucose; CR, complete response.

A 58-year-old man with a history of hepatitis B and type 2 diabetes mellitus presented at our hospital in March 2021 with right upper abdominal pain. Contrast-enhanced magnetic resonance imaging (MRI) revealed an enhanced solitary subcapsular lesion with a diameter of 1.5 cm in hepatic segment (S)6 (**Figure 1A**) that was radiologically suspected to be Barcelona Clinic Liver Cancer (BCLC) stage A1 HCC. Laboratory evaluation showed that the patient’s complete blood count and basic metabolic profile were within normal limits. In addition, laboratory testing revealed the following parameters: 16.6 µmol/L of total bilirubin, 18.3 U/L of alanine transaminase, 20.2 U/L of aspartate aminotransferase, 45.2 g/L of albumin, 1.5 ng/mL of α-fetoprotein, and a prothrombin time of 12.2 s. Hepatitis B viral DNA was not detected. The patient received transcatheter arterial chemoembolization (TACE) (with 20 mg of CalliSpheres^®^ beads [100–300 µm in diameter], 20 mg of lobaplatin, and 4 mL of lipiodol) and radiofrequency ablation (RFA), which resulted in a complete radiological response according to the Response Evaluation Criteria in Solid Tumors (version 1.1) (RECIST 1.1) (**Figure 1B**) (10). At this point, the laboratory tests again revealed nothing unusual. Contrast-enhanced MRI showed an enhanced small nodule with a diameter of 0.6 cm at the same location as the original tumor, and multiple enlarged ALNs (**Figures 1C**, **2A**) on July 26, 2021, indicating that the cancer had progressed to BCLC stage C. The patient subsequently underwent laparoscopic resection of the recurrent lesion in the S6 of liver and the lymph nodes in the hilar area. The pathology indicated the recurrent lesion as poorly differentiated high-grade HCC (**Figure 3**). The patient refused systemic therapy, including targeted therapy and chemotherapy, after the surgery. Two months later, a contrast-enhanced MRI showed no active lesions in the liver (**Figures 1D**, **2B**). However, positron emission tomography-computer tomography (PET-CT) detected increased glucose metabolism in two subcapsular lesions in the S5/S6 of the liver, with a maximum standardized uptake value (SUVmax) of 5.6 and a maximum size of 1.5×1.7× 1.8 cm and multiple enlarged ALNs (SUVmax = 9.3), with the largest measuring approximately 2.4×2.9×2.8 cm. These findings implied cancer recurrence (**Figure 4**). TACE was unavailable because of no obvious tumor staining during angiography. Hence, RFA of the lesions in the S5/S6 of the liver was performed owing to the results of the PET-CT. After the surgery, the patient was started on immunotherapy with tislelizumab (200 mg every 3 weeks). After four treatment cycles, contrast-enhanced MRI showed a complete response with no increase in local tumor size (in the arterial phase) or new lesion and a significant reduction in the size of the ALNs (≤10 mm) (**Figures 1E**, **2C**) assessed by RECIST 1.1. Although the patient experienced fatigue and developed skin rashes with pruritus over the entire body, he was cured by symptomatic treatment. The patient was maintained on regular medication until December, 2021, and contrast-enhanced MRI was performed every 3 to 6 months. By January 9, 2023, the patient had been in complete remission for ~15 months without evidence of tumor relapse (**Figures 1F**, **2D**). The patient experienced a temporary increase in C-reactive protein (CRP) levels (10.1 mg/L) but returned to within the normal range (0-10 mg/L), likely as a result of RFA treatment. During and after the treatment of tislelizumab, the blood test showed normal liver function and no sign of inflammation (**Table 1**). The patient’s treatment timeline is shown in **Figure 5**.

**Table 1:** Summary of liver function and inflammatory factors testing in the patient before and after the treatment of tislelizumab.

DATE	ALT(U/L)	AST(U/L)	TBIL (umo/L)	WBC (x10^9^/L)	NE (×10^9^L)	CRP (mg/L)	PCT (ng/mL)
2021-03-03	18.3	20.2	16.6	5.76	4.08		
2021-04-21	15.5	14.5	16.6	5.19	3.47		
2021-07-22	12.1	18.3	16.4	6.44	3.65	2.5	
2021-10-09*	16.1	18.8	20	5.8	3.94	10.1	
2021-11-10	17.1	22.5	11.3	3.8	2.08	1.1	0.033
2021-11-29	18.7	21.8	19	4.37	2.66	1.5	0.046
2021-12-20	36	27.2	17.1	4.92	2.49	1.9	
2022-02-09	25.8	24.9	21.3	4.87	3.28	1.8	
2022-03-29	30	23.4	16.3	5.11	2.82	1.25	

*RFA plus tislelizumab started on this day. ALT, alanine aminotransferase; AST, aspartate transaminase; TBIL, total bilirubin; WBC, white blood cell counts; NE, neutrophilicgranulocyte; CRP, C-reactive protein; PCT, procalcitonin.

## Discussion

According to current guidelines, systemic therapy with multikinase inhibitors (MKIs), which target the anti-vascular endothelial growth factor, are recommended for patients with advanced HCC (BCLC stage C) (11). Sorafenib has been approved as the only MKI for systemic therapies of HCC for ~10 years (12). However, sorafenib prolonged the median survival of advanced HCC patients by less than 3 months, and had an objective response rate (ORR) of less than 5% (12, 13). In 2018, another MKI, lenvatinib, which demonstrated noninferiority to sorafenib, became a valid alternative to sorafenib as a first-line therapy for HCC (14). Unfortunately, sorafenib and lenvatinib are usually associated with disease resistance or significant toxicity.

Recently, cancer immunotherapy has become the primary treatment option and has been approved by the FDA for the treatment of patients with advanced HCC. HCC may evade the immune system *via* the expression of immune checkpoint molecules such as PD-1, which is also expressed by activated T cells, B cells, natural killer cells, and myeloid cells. PD-1 interacts with programmed death ligand 1 (PD-L1) to repress the T-cell response against cancer cells (15). Thus, ICIs such as those targeting PD-1 or PD-L1, activate the immune system to eliminate tumor cells (16). The PD-1 inhibitors, nivolumab and pembrolizumab, have been used as a salvage therapy for patients with metastatic HCC who progressed during or after sorafenib treatment. However, nivolumab failed to significantly increase the median overall survival (OS) of HCC patients (for its predefined statistical threshold) in the phase III ChecMate459 trial (17). Meanwhile, pembrolizumab failed to demonstrate statistically significant superiority over placebo in terms of OS and progression-free survival in the phase III KEYNOTE-240 trial (18). Combination therapy using MKIs and ICIs such as atezolizumab (a PD-1 inhibitor) and bevacizumab (a MKI) showed superiority to sorafenib in terms of survival benefits and reduced toxicity, based on the results of the IMbrave 150 trial (19). Liu et al. reported a case of massive HCC with portal hepatic vein tumor thrombus and ALN metastases. The patient received atezolizumab plus bevacizumab and experienced a CR after three cycles of treatment (20). However, an optimal immunotherapeutic strategy for HCC has not been found.

In this study, the efficacy of combination therapy was not evaluated because the patient refused chemotherapy. Tislelizumab, an anti-PD-1 antibody developed in China that has a different structure to that of traditional anti-PD-1 antibodies (21), produced an ORR of 13% (32/249) in HCC patients regardless of the number of prior lines of treatment (22). Chao et al. reported a case of massive HCC without distal metastases in which the patient exhibited a CR and was downstaged for salvage resection after the combination treatment of TACE and tislelizumab. The patient maintained no recurrence 6 months after the treatment (23). The encouraging results of these previous studies led us to treat our patient with tislelizumab. Moreover, as a domestic product, tislelizumab is much cheaper than other ICIs, thus reducing the financial burden on the patient. In this case, the patient was diagnosed with HCC with multiple ALN metastasis but was able to remain free from cancer recurrence for up to 15 months because of locoregional therapies and tislelizumab treatment. The favorable results suggest that tislelizumab is a feasible monotherapy for HCC treatment. A large, global, phase III clinical trial (NCT03412773) has been performed to further evaluate the efficacy and safety of tislelizumab compared with sorafenib as a first-line treatment in adult patients with unresectable HCC (24).

Locoregional therapies, including RFA and TACE, have been shown to induce a peripheral immune response. This supports the use of ICI and locoregional therapy as a combination treatment for aggressive intermediate or advanced stage HCC (25). The combination of tremelimumab (an antibody targeting cytotoxic T-lymphocyte-associated protein 4) with locoregional therapies (e.g., TACE and RFA) has been evaluated in a phase II study including 32 patients with advanced HCC (25% BCLC stage B; 75% BCLC stage C) (26). 26% of the evaluable patients had a confirmed partial response, and a median time to progression (TTP) and OS of 7.4 and 12.3 months, respectively. We surmise that the potent antitumor effect could not be achieved by locoregional therapy or tislelizumab alone. As a result, we considered it rational to combine these two therapeutic strategies for the treatment of our patient. To the best of our knowledge, this is the first reported case of a complete response to anti-PD-1 immunotherapy and locoregional therapies in a patient with advanced HCC (BCLC stage C).

In summary, we encountered a case of advanced HCC with multiple ALN metastases, which were successfully (the patient showed a CR and no tumor recurrence up to 15 months) treated with tislelizumab and locoregional therapies. Considering that no consensus has been reached regarding the best immunotherapeutic options for HCC and that the efficacy of tislelizumab has not been extensively evaluated, our findings suggest that tislelizumab may serve as an effective monotherapy for HCC. Moreover, our results demonstrate that combining tislelizumab with locoregional therapies increases the efficacy of each therapy mode alone in the treatment of HCC.

## Data availability statement

The original contributions presented in the study are included in the article/supplementary material. Further inquiries can be directed to the corresponding author.

## Ethics statement

Ethical review and approval was not required for the study on human participants in accordance with the local legislation and institutional requirements. The patients/participants provided their written informed consent to participate in this study. Written informed consent was obtained from the participant/patient(s) for the publication of this case report.

## Author contributions

HD and BC contributed equally to this work. The treatment plan was designed by ZX, FP, and DP. Patient treatment was performed by FP, HD, and BC. HD, FP, and BC wrote the manuscript. FP, BC, JH, WZ and TC participated in data acquisition and manuscript revision. ZX, HD, and FP supervised the study. All authors contributed to the article and approved the submitted version.
